# Third Body Wear of an All-Polymer, PEEK-OPTIMA™ on Ultra-High-Molecular-Weight Polyethylene Total Knee Replacement

**DOI:** 10.3390/bioengineering12030261

**Published:** 2025-03-05

**Authors:** Raelene M. Cowie, Jens Schwiesau, Thomas M. Grupp, Adam Briscoe, Louise M. Jennings

**Affiliations:** 1 Institute of Medical and Biological Engineering, University of Leeds, Leeds LS2 9JT, UK; r.cowie@leeds.ac.uk (R.M.C.); abriscoe@invibio.com (A.B.); 2 Research & Development, Aesculap AG, 78532 Tuttlingen, Germany; jens.schwiesau@aesculap.de (J.S.); thomas.grupp@aesculap.de (T.M.G.); 3 Ludwig Maximilians University Munich, Department of Orthopaedic and Trauma Surgery, Musculoskeletal University Center Munich (MUM), Campus Grosshadern, LMU Munich, 81377 Munich, Germany; 4 Invibio Biomaterials Solutions, Lancashire FY5 4QD, UK

**Keywords:** knee, PEEK-OPTIMA, UHMWPE, third body wear, experimental simulation

## Abstract

Experimental wear simulation of a PEEK-OPTIMA™ polymer-on-UHMWPE total knee replacement has shown equivalent UHMWPE wear to conventional knee replacement materials (cobalt chrome-on-UHMWPE) when tested in a clean environment. The aim of this study was to experimentally investigate the wear of this all-polymer total knee replacement under third body wear conditions. Three PEEK-OPTIMA™ and three cobalt chrome femoral components articulating against all-polyethylene tibial components were tested in a knee simulator. One million cycles of wear simulation was carried out in clean lubricant under conditions replicating walking followed by one million cycles with the lubricant contaminated with porcine bone particles, then one million cycles with PMMA cement particles. UHMWPE wear was determined gravimetrically. Statistical analysis compared UHMWPE wear against PEEK femoral components to cobalt chrome. In clean lubricant and with bone particles in the lubricant, there was no significant difference (*p* > 0.05) in UHMWPE wear against the different femoral component materials, and wear rates were similar under both conditions. With PMMA particles, there was a dramatic increase in UHMWPE wear for both knee replacement systems but no significant difference (*p* > 0.05) in UHMWPE wear between the femoral component materials. This is the first study to investigate wear of an all-polymer knee under third body wear conditions.

## 1. Introduction

With over 100,000 procedures carried out in England, Wales, Northern Ireland, the Isle of Man and Guernsey annually and a >95% survivorship at 10 years and >90% at 20 years, total knee replacement is considered to be a highly successful procedure [1] (National Joint Registry 21st annual report Figure 3K3(a)). However, there is a cohort of patients who are dissatisfied with their knee replacement [2]. Recent studies have shown a larger number of patients than previously considered may react adversely to the metal in their knee replacement, so there is a move to investigate the potential for metal-free joint replacement systems [3,4,5]. PEEK-OPTIMA™ (PEEK), a high-performance polymer, is one material under consideration as an alternative to cobalt chrome (CoCr) for the femoral component of total knee replacements, and when coupled with an UHMWPE (ultra-high-molecular-weight polyethylene) all-polymer tibial component, it provides a metal-free knee replacement system [3,6].

Wear simulation studies of natural (unfilled) PEEK-on-UHMWPE carried out to date have shown a similar rate of UHMPWE wear of the PEEK-on-UHMWPE bearing couple compared to CoCr-on-UHMWPE in both simple geometry wear simulation [7,8,9,10] and whole-joint knee simulation studies [3,5,6]. These studies have primarily been conducted under optimal alignment conditions and in clean lubricant. To fully understand how the knee replacement system will respond when implanted, a wider range of surgical alignment and environmental conditions to which the knee replacement system could be subjected to in the body should be considered. These methods will likely go beyond the optimised conditions currently applied in standardised laboratory test methods [11]. Third body wear is one condition under which the all-polymer knee replacement system has not yet been investigated.

To date, third body wear simulation of the UHMWPE-on-PEEK couple has been limited to studies in a simple geometric (pin-on-plate) configuration which showed (i) PEEK to be less resistant to scratching by hard particles such as PMMA (polymethyl methacrylate) bone cement (with ZrO_2_ as a radiopacifier) than cobalt chrome and (ii) that when severe scratches were made in PEEK (lip height ~4 µm), there was a polishing effect of the PEEK by the UHMWPE, and no increase in UHMWPE wear was measured compared to unscratched controls; similar findings did not occur in the UHMWPE-on-CoCr bearing couple [8]. Whether these findings translate to the whole-joint knee simulation model is unknown.

There are a number of challenges associated with carrying out third body wear simulation, particularly of total knee replacements where particles may be ejected from the interface of the femoral and tibial components during the test. In previous studies, this has been overcome by running the total knee replacement with the third body particles in situ without lubricant to embed the particles prior to adding lubricant and continuing the wear test [12,13]; however, this technique has not been adopted in all investigations [14]. There are potential issues associated with embedded particles influencing gravimetric wear measurements [15,16], and when selecting particles for third body wear simulation, consideration should be given to the hardness of the particle, its composition, size and shape to give the test method clinical relevance [16]. PMMA cement particles have been identified both in the joint following implantation and embedded in polyethylene of retrieved knee and hip devices [17,18,19,20] and have been commonly used to simulate third body wear conditions. In vitro investigations of metal-on-polyethylene implants have shown that to accelerate wear, there is a need for the PMMA cement to contain radiopacifiers, either zirconium dioxide or barium sulphate, which have the potential to form hard agglomerates, scratching the metal counterface and ultimately accelerating polyethylene wear [21,22,23]. Bone particles are also commonly found in retrieved metal-on-polyethylene knee replacements [19,24]; however, despite having been shown to deform polyethylene, studies have demonstrated that when trapped between the articulating surfaces of a joint, the resulting scratches on a metal counterface do not have sufficiently high scratch lip heights to accelerate UHMPWE wear [8,14,21,22,23,25]. These findings are likely due to the hardness of the bone particles being insufficiently high to result in severe abrasion of a metal counterface. Scratching occurs when the hardness of a substrate is less than 80% of that of the third body particles present in the joint [16,26]. The wear of the PEEK-on-UHMWPE knee replacement under third body wear conditions when challenged with bone and PMMA cement particles is unknown.

The aim of this study was to investigate the wear of an all-polymer, PEEK-OPTIMA-on-UHMWPE total knee replacement under third body wear conditions with cortical bone particles and PMMA cement particles. The wear of the all-polymer knee was compared to a CoCr-on-UHMWPE knee of similar initial surface topography and geometry. It was hypothesised that the lower hardness of the PEEK femoral component would lead to more scratching and a greater increase in surface roughness compared to CoCr but that the increase in surface roughness would not lead to an increase in UHMWPE wear compared to CoCr. This is the first study to investigate the wear of an all-polymer PEEK-on-UHMWPE knee replacement system under third body wear conditions.

## 2. Materials and Methods

Three size C (mid-sized), cruciate-retaining cobalt chrome femoral components (Maxx Freedom Knee, Maxx Medical, Norristown, PA, USA) and 3 size C, cruciate-retaining PEEK-OPTIMA™ femoral components (Invibio Knees Ltd., Lancashire, UK) [3,6] of similar initial surface topography and geometry coupled with 6 GUR 1020 all-polyethylene tibial components (Maxx Medical, Norristown, PA, USA) were investigated. In addition, 2 identical UHMWPE tibial components were used as unloaded soak controls to compensate for moisture uptake by the polyethylene during the investigation. The polyethylene was not cross-linked and was sterilised with ethylene oxide prior to the study. The femoral components were set up on the distal centre of rotation consistent with previous investigations of this knee replacement system [3,6] using custom fixtures. Tibial components were cemented with their paired femoral components in such a way that they could be removed from the fixtures for analysis. Prior to the start of the study, the tibial components were soaked in sterile water (minimum 2 weeks) to maximise their moisture uptake.

The third body particles of interest were porcine cortical bone obtained from the femurs of 6-month-old white pigs and Palacos R&G PMMA cement particles (Heraeus Medical GMBH, Wehrheim, Germany). Although bone particles have been previously shown to have no influence on the wear of CoCr-on-UHMWPE in knee simulation studies [14,27], they were investigated in this study due to the high potential for bone to remain in the joint following surgery and subsequent lavage [19] and because the influence of bone particles on the wear of the PEEK-on-UHMWPE bearing couple is unknown. Porcine bone was chosen over human for ethical purposes, but it is acknowledged that the modulus of porcine bone is lower than that of human bone [28], and therefore the particles may not be as abrasive as those generated during implantation [19] or a highly calcified human osteophyte for example [29]. The PMMA cement used was the same as that used for fixation of the device and contained zirconium dioxide as a radiopacifier. The cement was mixed and allowed to cure as per the manufacturer’s instructions; particles were prepared by grinding the cement (using a bladed grinder) which had been frozen at −20 °C and subsequently sieving to a size range between 500 and 1000 µm. Bone particles were prepared in a similar manner by dissecting cortical bone, freezing, grinding and subsequently sieving. Despite similar preparation of the two particle types, there were differences in both the size and morphology of the particles. Both particle types had a granular, irregular geometry, but with the PMMA particles generally being larger and rounder than the bone particles (Table 1). The aim of the study was to compare the wear of the all-polymer knee to a metal-on-polyethylene knee replacement so differences in the particle size and shape had no influence on the study hypothesis. The size range and particle concentration were chosen based on the particle size used in previous investigations, which has been shown to increase wear in a metal-on-polyethylene knee joint [14,30].

**Figure 1:**
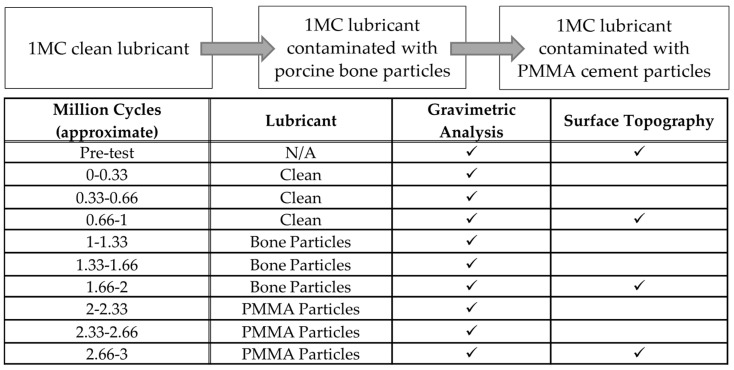
Experimental design and the measurement points.

The studies were carried out on a 6-station ProSim electromechanical knee simulator (Simulation Solutions Ltd., Stockport, UK), which was calibrated prior to use. The simulator has 4 controlled axes of motion: axial force (AF), flexion/extension (FE), anterior–posterior translation (AP) and tibial rotation (TR). The medial–lateral (ML) translation was fixed, and the abduction/adduction (AA) rotation was unconstrained. The anterior–posterior translation and tibial rotation were delivered through the tibial component and run under Leeds high-kinematic displacement-controlled conditions [31]. A maximum axial force of 2800 N, a flexion/extension range of 0–58°, an anterior–posterior displacement of up to 10 mm and a tibial rotation of ±5° were used (Figure 1). The axial force was offset 7% of the width of the tibial component in the medial direction to give greater load sharing in the medial compartment [32,33]. The cycle frequency was 1 Hz. The simulators can run with the anterior–posterior displacement and tibial rotation axes in either force or displacement control modes, which are chosen based on the research question. For this study into third body wear, displacement control was chosen so that the interaction between the implants and third body particles was the only variable under investigation [34]. Simulator running temperature conditions were used to minimise test artefacts such as protein degradation and precipitation, which may artificially lower UHMWPE wear. This approach has been optimised for the PEEK-on-UHMWPE bearing couple and is consistent with previous wear simulation of the all-polymer total knee replacement [6,7].

The experimental design is shown in Figure 1 and was consistent with previous third body wear simulation studies [14]. Initially, the knee replacements were run for 1 million cycles (MC) in clean lubricant (25% newborn calf serum diluted with 0.03% (*v*/*v*) sodium azide solution to give a protein concentration of 16 g/L) to determine the baseline wear rates. Every 0.3 MC, the simulator was cleaned, the wear of the UHMWPE tibial components was determined by gravimetric analysis and the simulator was reassembled with clean lubricant. After 1 million cycles of simulation in clean lubricant, the influence of third body wear with bone particles was investigated. The concentration of particles used (5 mg/mL) is consistent with previous investigations in metal-on-polyethylene knee replacements and has been shown to be a sufficiently high concentration of PMMA particles to accelerate UHMWPE wear [14,30]; in this study, the same concentration of particles was used irrespective of the particle composition. A total of 2.5 g of bone particles were added to the test cell ensuring the surface of the tibial component was covered with particles before assembling the knee and adding 500 mL of lubricant. Then, 1 MC of wear simulation was conducted with the lubricant contaminated with bone particles. After each 0.3 MC, the test cell and components were cleaned, gravimetric analysis of the tibial components was carried out then the test cell was reassembled and the particles replenished. Adding new particles at each serum change gave a worst-case scenario and was considered appropriate in this study because the particles used when entering the joint space in the body were not anticipated to degrade [12,13]. Following wear simulation with porcine bone particles, an additional 1 MC of wear simulation was carried out with the lubricant contaminated with PMMA cement particles (5 mg/mL). Gravimetric analysis and replenishment of particles was carried out every 0.3 MC.

Gravimetric analysis of the UHMWPE tibial components was carried out on an XP205 digital microbalance (Mettler Toledo, Leicester, UK) with a resolution of 10 mg. Measurements were taken before the start of the study to set a datum then after every 0.3 MC of wear simulation (Figure 1). Prior to weighing, components were cleaned ultrasonically in 70% propan-2-ol before being allowed to stabilise (minimum 48 h) before weight measurements were taken. Loss in mass of the UHMWPE was converted to a wear volume using a density of 0.938 g/cm^3^ for GUR 1020 UHMWPE. The surface topography of the femoral and tibial components was measured prior to the start of the study then after every subsequent 1 MC (at each change of test condition) using a PGI 800 contacting Form Talysurf (Taylor Hobson, Leicester, UK). Prior to taking measurements, the condition of the stylus was checked and verified against a standard of known roughness. On the femoral components, 5 measurements were taken per condyle in the flexion range of the study in a medial–lateral direction; on the tibial components, 4 measurements were taken on both the medial and lateral compartments within the contact region in a medial–lateral direction. Form removal and filtering were used appropriate to the material of interest. The mean surface roughness (Ra) of the femoral and tibial components has been shown with further analysis of the articulating surfaces in the University of Leeds data repository [35]. Images of the articulating surfaces were taken using an Alicona G5 optical microscope with 10× magnification. The bulk lubricant temperature was measured every 0.3 MC by inserting a thermocouple (Fluke 52II, Everett, DC, USA) into the lubricant in the test cell close to the articulating surfaces.

The mean wear rate, mean surface roughness (Ra) and bulk lubricant temperature were calculated with 95% confidence limits. Statistical analysis was carried out using Microsoft Excel and a one-way ANOVA to compare the PEEK and CoCr knee replacement systems with significance taken at *p* < 0.05. Data associated with this study are available through the University of Leeds data repository [35].

## 3. Results

Prior to the start of the study, there was no significant difference in the mean surface roughness of the PEEK and CoCr femoral components (*p* > 0.05), or the tibial components articulating against the different femoral component materials (*p* > 0.05) (Table 2).

**Figure 2:**
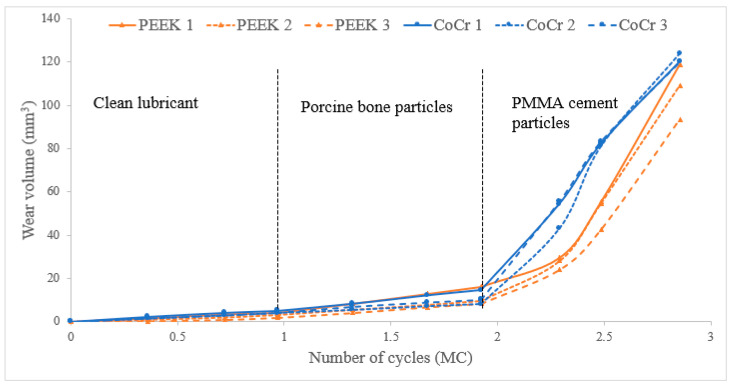
Wear volume of UHMWPE tibial components (mm^3^) against CoCr and PEEK femoral components during the three phases of the study.

**Figure 3:**
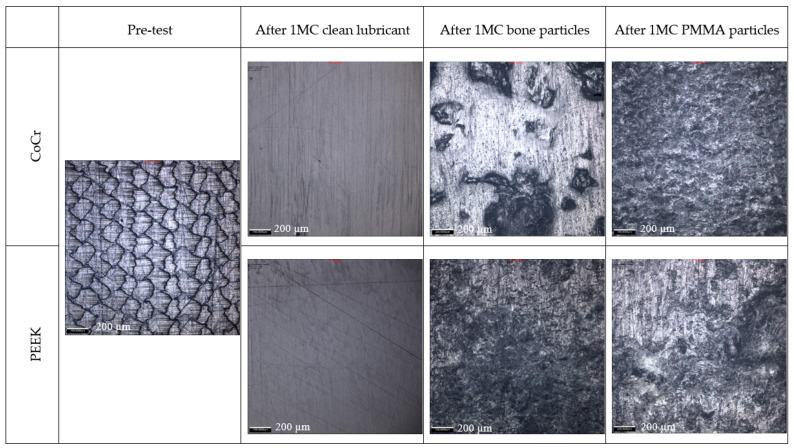
Representative images of UHMWPE tibial components (medial compartment, middle of wear scar) articulating against CoCr and PEEK femoral components taken with 10× magnification using an Alicona G5 optical microscope; scale bar represents 200 µm.

**Figure 4:**
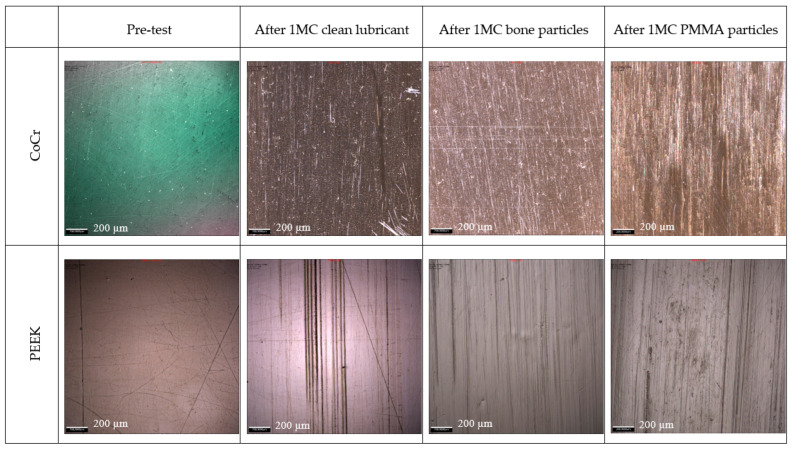
Representative images of CoCr and PEEK femoral components (centre of medial condyle at 30° flexion) taken with 10× magnification using an Alicona G5 optical microscope; scale bar represents 200 µm. Note: The measurement taken for the CoCr pre-test was taken under different lighting conditions; the difference is only in the colour of the image.

After 1 MC of wear simulation in clean lubricant, the mean wear rate of the UHMWPE tibial components was 4.5 ± 1.5 and 3.2 ± 2.9 mm^3^/MC articulating against CoCr and PEEK, respectively. There was no significant difference in the wear rate of the UHMWPE tibial components against the two femoral component materials, *p* = 0.15. The wear volume of each tibial insert over the duration of the study is shown in Figure 2. On the tibial components, polishing was evident where the machining marks on the tibial components had been removed (Figure 3); there was some evidence of light scratching running in an anterior–posterior direction on the PEEK femoral components (Figure 4) and isolated light scratches on the CoCr femoral components. After 1 MC, the mean surface roughness for both materials increased and the Ra of the PEEK femoral components was significantly higher compared to the CoCr femoral components, *p* = 0.01 (Table 2). For the metal-on-polyethylene knee replacements, the bulk lubricant temperature was similar to the soak control; for the all-polymer knee, the bulk lubricant temperature was higher than the soak control and significantly higher than the conventional materials, *p* = 0.001 (Table 3).

**Figure 5:**
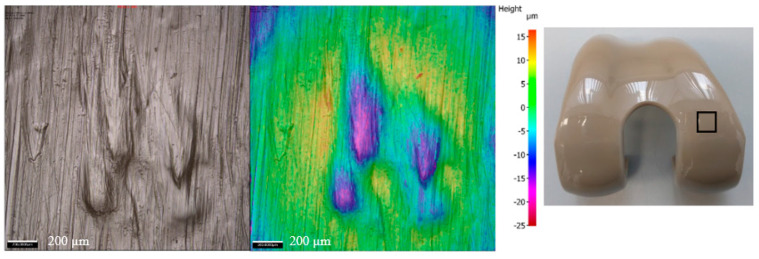
Images of PEEK femoral components taken at 10× magnification using an Alicona G5 optical microscope showing the deformation which occurred at full extension following simulation with porcine bone particles. Scale bar represents 200 µm; the image on the right shows the approximate region in which the images were taken on an unworn implant.

Following 1 MC of wear simulation with porcine bone particles, there was visible pitting in the surface of the tibial components and evidence of embedded particles in the UHMWPE (Figure 3). The surface of the UHMWPE articulating against PEEK and CoCr was visibly different with the whole of the surface articulating against the PEEK femoral component appearing abraded, and against the CoCr femoral component, polished regions were interspersed with pits. There was, however, no significant difference in the mean surface roughness of the tibial components articulating against the different femoral component materials, *p* = 0.238. There were no visible changes on the surface of the CoCr femoral components, and the surface roughness measurements were similar to those carried out following wear simulation in clean lubricant; the PEEK femoral components however had a high density of linear scratching on the articulating surface and also evidence of deformation in full extension where high loads are applied during the simulation for all the PEEK femoral components (Figure 4 and Figure 5). The scratching on the PEEK femoral components resulted in a significantly higher (*p* < 0.001) mean surface roughness of the PEEK femoral components compared to CoCr (Table 2). When challenged with porcine bone particles, after 1 MC wear simulation, the wear rate of the UHMWPE tibial components was higher than when tested in clean conditions with a wear rate of 6.9 ± 7.1 and 8.8 ± 8.5 mm^3^/MC against CoCr and PEEK, respectively; however, there was no significant difference in UHMWPE wear against the different femoral component materials, *p* = 0.50. The bulk lubricant temperature was similar to when testing was carried out in clean lubricant, with the temperature of the all-polymer bearing couple being significantly higher than the metal-on-polyethylene knee, *p* < 0.001 (Table 3).

**Figure 6:**
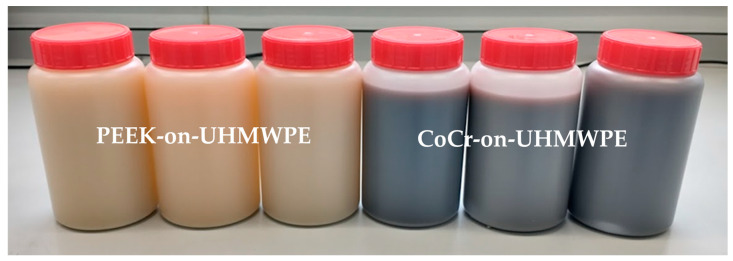
Lubricant samples from the PEEK-on-UMWPE knee (**left**) and the CoCr-on-UHMWPE knee replacement (**right**) following 0.3 MC of wear simulation with PMMA cement particles added to the lubricant. Note the discolouration of the lubricant in the CoCr-on-UHMWPE test.

When PMMA particles were introduced into the test cell, the lubricant in the CoCr-on-UHMWPE study turned from straw-coloured to black; there was no visible change in the lubricant in the all-polymer knee replacement system (Figure 6). On the UHMWPE tibial components articulating against CoCr, the whole of the contact surface appeared abraded; against PEEK, there appeared to be polishing of the abraded areas (Figure 3). Against both materials, the Ra of the tibial components increased (Table 2), but there was no significant difference in the mean surface roughness of the tibial components articulating against the different femoral component materials, *p* = 0.350. PMMA particles were observed embedded in the polyethylene articulating against both femoral component materials. On the femoral components, scratching was visible on both PEEK and CoCr femoral components (Figure 4), the Ra of the CoCr femoral components was higher than following the study with porcine bone particles and polishing of the PEEK femoral components occurred, which lowered their mean surface roughness (Table 2). There was no significant difference in Ra between the two femoral component materials, *p* = 0.058. With the introduction of PMMA cement particles into the lubricant, the wear rate of the UHMWPE increased >10-fold for both material combinations with a mean wear rate of 120.4 ± 16.0 and 103.7 ± 26.4 mm^3^/MC for the metal-on-polyethylene and all-polymer knee replacements, respectively. However, there remained no significant difference in UHMWPE wear rate between the two materials, *p* = 0.08. The bulk lubricant temperature in the metal-on-polyethylene knee replacement system was higher than in the phases of the study carried out in clean lubricant and porcine bone particles and was similar (*p* = 0.68) to the temperature of the test with the all-polymer knee (Table 3). Over the duration of the study, the lubricant temperature in the soak control remained consistent at between 25.8 and 26.1 °C.

## 4. Discussion

There are several potential advantages of using PEEK-OPTIMA™ as the femoral component of total knee replacements, and experimental studies have shown equivalent wear of the all-polymer knee replacement system compared to a similar metal-on-polyethylene design when tested in clean lubricant [3,6]. However, the behaviour of the all-polymer knee replacement under third body wear conditions has not previously been investigated. The aim of this study was to investigate the wear of the all-polymer knee when particles of porcine bone and PMMA cement were introduced into the test cell and to compare the wear of the all-polymer knee to that of a metal-on-polyethylene total knee replacement system of similar initial surface topography and geometry.

Wear simulation in clean lubricant (without contaminants) resulted in a similar (*p* = 0.15) wear rate to previous investigations carried out under displacement-controlled, Leeds high-kinematic conditions with wear rates of 3.2 ± 2.9 and 4.5 ± 1.5 mm^3^/MC for the all-polymer and metal-on-polyethylene knee replacements, respectively [6]. In addition, the higher bulk lubricant temperature measured in the all-polymer device compared to the metal-on-polyethylene when tested in clean lubricant has been previously reported, thought to be as a result of higher friction of the all-polymer bearing couple resulting in frictional heating [3,7].

Bone fragments generated during the implantation procedure are commonly found in the joint following total knee arthroplasty procedures [19]. In this study, porcine cortical bone fragments were used to simulate the presence of bone debris in the joint. Previous studies introducing bone particles into the lubricant of metal-on-polyethylene total knee prostheses have shown deformation of the UHMWPE tibial components [24] and particles becoming embedded in the polyethylene but no acceleration in wear [14,24]. The findings from this study reflect those from previous investigations for metal-on-polyethylene knee replacements [14,27]. For the all-polymer knee, scratching was also visible on the surface of the PEEK femoral components; this was anticipated as previous investigations in simple geometry have shown PEEK to be less resistant to scratching than CoCr [8]. There was also evidence of deformation on the PEEK femoral components likely caused by the bone particles. However, no significant difference in wear rate between the UHMWPE tibial components articulating against PEEK or CoCr was measured.

Investigations into third body wear with PMMA cement have shown that without a radiopacifier, addition of PMMA cement into a metal-on-polyethylene interface neither scratches the metal nor accelerates polyethylene wear. However, the inclusion of radiopacifiers into PMMA can be highly abrasive, scratching metal surfaces and accelerating wear. Zirconium dioxide has been shown to increase UHMWPE wear to a greater extent than barium sulphate due to the formation of larger agglomerates [29,36]. In this study, zirconium dioxide was the radiopacifier used in the PMMA cement and, consistent with previous investigation of metal-on-polyethylene bearings, caused scratching on the cobalt chrome counterface, resulting in a >10-fold acceleration of UHMWPE wear. A similar >10-fold increase in UHMWPE wear was also observed in the all-polymer knee. For both material combinations, the UHMWPE wear rate was linear over the duration of the million cycles where the lubricant was contaminated with bone cement likely in part due to the replenishment of particles at each serum change. The approach of replacing particles replicated the extended duration the PMMA particles would be present in the joint (as they are non-degradable) and created a worst-case scenario, so at each measurement point, there was a high availability of highly abrasive particles to become trapped in the articulating surfaces and further accelerate wear.

Discolouration of the lubricant observed in the metal-on-polyethylene study suggests the release of metal particles from the CoCr femoral component surface into the lubricant. This finding has previously been reported in experimental simulation of third body wear in metal-on-metal hips when metal debris was introduced into the lubricant creating a highly abrasive, high metal wear scenario [37]. Metal particles or ions may be released due to wear or corrosion and may have local and systemic effects [38]. Case studies have reported early metallosis-related failure primarily occurring when metal-on-metal contact occurs in the joint; however, metal hypersensitivity reactions to metal-on-polyethylene total knee replacements are difficult to predict pre-operatively, for example through patch testing, and post-operatively, they can be difficult to differentiate from other acute reactions to the joint or skin. Therefore, they are likely under-diagnosed and under-reported [39,40,41,42,43,44,45]. Much of the current research investigating the response of patients to metal ions and debris has focused on patients with metal-on-metal hip prostheses. Local reactions such as pseudotumours are frequently reported [46], as well as a decrease in osteoblast proliferation, which may have a role in implant failure due to osteolysis [47]. Systemic toxicity due to chronic exposure to metal ions from metal-on-metal hip implants has been observed but is extremely rare and associated with cardiac, sensory and neurological changes in the patient [46,48]. PEEK, however, is relatively bioinert; an in vitro study comparing PEEK particles to UHMWPE particles has demonstrated a reduced macrophage cytokine response to PEEK compared to UHMWPE [49].

Discolouration of the lubricant was not observed in the all-polymer knee replacement. It is likely that wear of the PEEK occurred in this study, due to the harshness of the test protocol, which used a high concentration of abrasive particles that were replenished at each measurement point. However, PEEK wear could not be assessed. The inconsistent moisture uptake of PEEK makes assessing wear gravimetrically unreliable, and geometric methods for assessment of femoral condyles are limited with no standardised methods currently available. When PMMA particles were added to the test cell, the bulk lubricant temperature of the metal-on-polyethylene knee replacement was also higher than under conditions using clean lubricant and when simulated with bone particles. Similar findings were not evident for the all-polymer bearing couple and suggest that with PMMA cement contamination, there was an increase in the friction for the metal-on-polyethylene combination.

There are a number of limitations associated with this study which may provide the basis for future investigations, including the sample size. Only three knee replacement systems of each material combination were investigated due to the number of stations in the simulator (six) and the necessity to carry out the investigation of the all-polymer and the metal-on-polyethylene knee replacements in parallel. In addition, each condition was only investigated for 1 million cycles, whilst this is a relatively short duration, it was sufficient to observe clear trends in the wear of the different materials. Assessing wear of the UHMWPE tibial components gravimetrically is also a limitation as particles embedded in the polyethylene have the potential to mask wear [16]. It was important to use the same concentration of particles for both material combinations, so the likelihood of particles being embedded in the polyethylene was the same for both implant types. Whilst the concentration of the particles was matched to previous investigations, there are a number of unknown parameters which could influence the availability of particles to become trapped between the articulating surfaces and make comparing the results of this study to previous investigations difficult, including the mass of the particles used, the volume of lubricant and whether the lubricant was static in the test cell or recirculated. The discolouration of the lubricant in the metal-on-polyethylene knee replacements when using PMMA cement particles and the high degree of scratching on the PEEK femoral components suggest the possibility of material loss from the femoral components; however, this could not be quantified. Currently, there is no standardised test method for the geometric assessment of UHMWPE wear in the knee, nor is there a standardised method for the assessment of wear on the femoral component.

## 5. Conclusions

The results of this study confirm the hypothesis that under third body wear conditions with bone particles and bone cement particles, the lower hardness of the PEEK femoral component led to scratching and a greater increase in the surface roughness of the femoral component compared to CoCr; however, the increase in surface roughness of the PEEK femoral component did not lead to an increase in UHMWPE wear compared to CoCr. There was no significant difference in the wear rate of the UHMWPE tibial components against the two femoral component materials for any of the conditions investigated (clean conditions, *p* = 0.15; bone particles, *p* = 0.50; PMMA, *p* = 0.08).

## Figures and Tables

**Table 1:** Mean diameter (µm) and aspect ratio ± standard deviation of porcine bone and PMMA cement particles generated for use in this study.

	Porcine Bone Particles	PMMA Cement Particles
Mean diameter (µm)	696.3 ± 257.0	988.3 ± 242.5
Mean aspect ratio	1.68 ± 0.70	1.30 ± 0.34

**Table 2:** Mean surface roughness (Ra) of femoral and tibial components (±95% CL), *n* = 3. Additional surface roughness data included in supplementary data [35].

Test Condition	Femoral Components			Tibial Components		
	CoCr	PEEK	Significance	CoCr	PEEK	Significance
Pre-test	0.022 ± 0.009	0.021 ± 0.004	*p* = 0.65	1.062 ± 0.057	1.091 ± 0.028	*p* = 0.124
Clean lubricant	0.039 ± 0.075	0.131 ± 0.054	*p* = 0.01	0.524 ± 0.275	0.660 ± 0.142	*p* = 0.131
Porcine bone particles	0.027 ± 0.006	0.398 ± 0.131	*p* < 0.001	8.442 ± 5.449	6.645 ± 1.182	*p* = 0.238
PMMA cement particles	0.124 ± 0.001	0.234 ± 0.179	*p* = 0.058	12.569 ± 8.814	9.286 ± 10.045	*p* = 0.350

**Table 3:** Mean bulk lubricant temperature (±95% CL) of UHMWPE tibial components.

Test Condition	Bulk Lubricant Temperature (°C)			
	Unloaded Soak Control	CoCr Femoral Components	PEEK Femoral Components	Significance
Clean lubricant	25.8	26.2 ± 1.5	29.2 ± 0.2	*p* = 0.001
Porcine bone particles	26.1	26.4 ± 1.5	30.6 ± 1.0	*p* < 0.001
PMMA cement particles	26.1	30.7 ± 1.6	30.5 ± 1.2	*p* = 0.68

## Data Availability

The data associated with this article are openly available through the Leeds University data repository at https://doi.org/10.5518/1644 [35].
